# Supplementary material for: Aphid Colony Size in Tansy is Affected by Plant Chemical Composition but not by Belowground Herbivory
Source: J Chem Ecol. 2025 May 29;51(3):63. doi: 10.1007/s10886-025-01609-y (PMC12122609; doi:10.1007/s10886-025-01609-y)
Supplement: Supplementary file 1 — Supplementary file1 (DOCX 729 KB) [file 10886_2025_1609_MOESM1_ESM.docx]

SUPPLEMENTARY INFORMATION


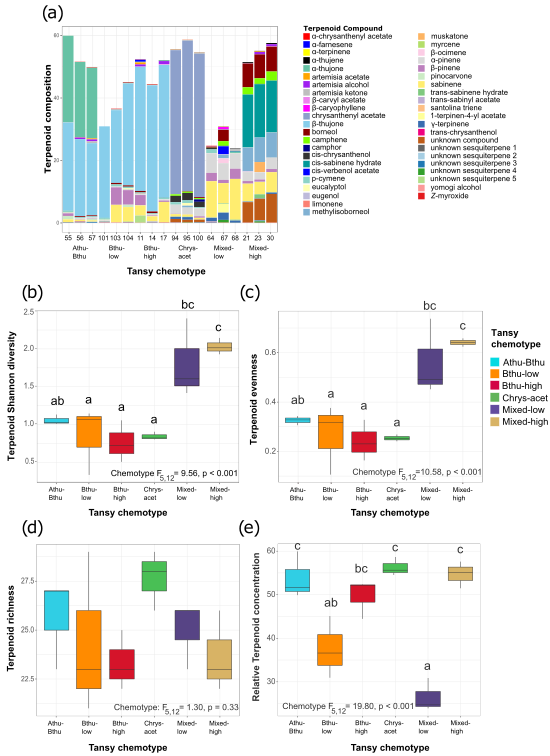


**Fig. 5**: Differences in chemical composition of leaves of different *T. vulgare* chemotypes. (a) Stacked bar chart showing the composition of the chemical profiles of each of the three daughters of the selected six maternal chemotypes. Box plots (b-e) show differences among chemotypes as interquartile ranges of (b) terpenoid Shannon diversity, (c) terpenoid evenness, (d) terpenoid richness, and (e) relative total terpenoid concentration for each chemotype. Chemotype effect is indicated in panels, letters above bars indicate significant differences (p<0.05) between chemotypes based on post-hoc Tukey tests. Boxplots represent n = 3 daughters per chemotype. This figure was taken from (Neuhaus‐Harr et al., 2024), by the same author, and is presented here for clarity.

**Table 4** OVERVIEW OF ALL 18 DAUGHTERS (THREE DAUGHTERS PER CHEMOTYPE), THEIR DOMINANT COMPOUND(S), THEIR TERPENOID SHANNON DIVERSITY, TERPENOID EVENNESS, TERPENOID RICHNESS AND TOTAL TERPENOID CONCENTRATION

| Chemotype | Daughter | Dominant compound | Shannon diversity | Even-ness | Rich-ness | Total conc. |
| --- | --- | --- | --- | --- | --- | --- |
| Bthu_low | 101 | β-thujone | 0.33 | 0.11 | 23 | 30.94 |
| Bthu_low | 103 | β-thujone | 1.15 | 0.38 | 21 | 36.61 |
| Bthu_low | 104 | β-thujone | 1.07 | 0.32 | 29 | 45.09 |
| Mixed_high | 21 | Mixed (borneol, cis-sabinene hydrate, eugenol, sabinene, others) | 1.93 | 0.62 | 22 | 51.48 |
| Mixed_high | 23 | Mixed (borneol, cis-sabinene hydrate, eugenol, sabinene, others) | 2.15 | 0.66 | 26 | 55.08 |
| Mixed_high | 30 | Mixed (borneol, cis-sabinene hydrate, eugenol, sabinene, others) | 2.03 | 0.64 | 23 | 57.60 |
| Chrys_Acet | 94 | Chrysantenyl acetate | 0.91 | 0.27 | 29 | 58.66 |
| Chrys_Acet | 95 | Chrysantenyl acetate | 0.80 | 0.24 | 28 | 54.52 |
| Chrys_Acet | 100 | Chrysantenyl acetate | 0.82 | 0.25 | 26 | 55.64 |
| Athu_Bthu | 55 | α-thujone; β-thujone | 1.03 | 0.33 | 23 | 59.94 |
| Athu_Bthu | 56 | α-thujone; β-thujone | 1.01 | 0.31 | 27 | 51.61 |
| Athu_Bthu | 57 | α-thujone; β-thujone | 1.13 | 0.34 | 27 | 49.90 |
| Bthu_high | 11 | β-thujone | 1.06 | 0.33 | 25 | 52.37 |
| Bthu_high | 14 | β-thujone | 0.51 | 0.16 | 22 | 44.44 |
| Bthu_high | 17 | β-thujone | 0.72 | 0.23 | 23 | 52.14 |
| Mixed_low | 64 | Mixed (sabinene, α-pinene, others) | 1.60 | 0.49 | 26 | 24.71 |
| Mixed_low | 67 | Mixed (sabinene, α-pinene, others) | 2.40 | 0.74 | 26 | 30.87 |
| Mixed_low | 68 | Mixed (sabinene, α-pinene, others) | 1.42 | 0.45 | 23 | 23.91 |

**Table 5** OVERVIEW OF ALL 240 PLANTS THAT WERE USED FOR THE EXPERIMENT. THREE DAUGHTERS PER EACH OF THE SIX CHEMOTYPES WERE USED. EACH DAUGHTER WAS USED EITHER THREE OR FOUR TIMES PER TREATMENT FOR EACH CHEMOTYPE, TOTALING TEN REPLICATES PER CHEMOTPYE. PLANTS WERE RANDOMLY DISTRIBUTED OVER TEN BLOCKS

| Chemotype | Daughter | ID | Aphids | Wireworms (WW) | Treatment | Block |
| --- | --- | --- | --- | --- | --- | --- |
| Bthu_high | 11 | 4 | Yes | No | Aphids | 1 |
| Bthu_high | 11 | 8 | Yes | Yes | Aphids + WW | 1 |
| Bthu_high | 11 | 5 | No | No | Control | 1 |
| Bthu_high | 11 | 18 | No | Yes | WW | 1 |
| Mixed_high | 23 | 17 | Yes | No | Aphids | 1 |
| Mixed_high | 23 | 2 | Yes | Yes | Aphids + WW | 1 |
| Mixed_high | 23 | 10 | No | No | Control | 1 |
| Mixed_high | 23 | 18 | No | Yes | WW | 1 |
| Athu_Bthu | 55 | 4 | Yes | No | Aphids | 1 |
| Athu_Bthu | 55 | 9 | Yes | Yes | Aphids + WW | 1 |
| Athu_Bthu | 55 | 14 | No | No | Control | 1 |
| Athu_Bthu | 55 | 15 | No | Yes | WW | 1 |
| Mixed_low | 68 | 10 | Yes | No | Aphids | 1 |
| Mixed_low | 68 | 17 | Yes | Yes | Aphids + WW | 1 |
| Mixed_low | 68 | 8 | No | No | Control | 1 |
| Chrys_Acet | 94 | 11 | Yes | No | Aphids | 1 |
| Chrys_Acet | 94 | 5 | Yes | Yes | Aphids + WW | 1 |
| Chrys_Acet | 94 | 19 | No | No | Control | 1 |
| Chrys_Acet | 94 | 4 | No | Yes | WW | 1 |
| Bthu_low | 103 | 9 | Yes | No | Aphids | 1 |
| Bthu_low | 103 | 14 | Yes | Yes | Aphids + WW | 1 |
| Bthu_low | 103 | 8 | No | No | Control | 1 |
| Bthu_low | 103 | 7 | No | Yes | WW | 1 |
| Bthu_high | 17 | 13 | Yes | No | Aphids | 2 |
| Bthu_high | 17 | 7 | Yes | Yes | Aphids + WW | 2 |
| Bthu_high | 17 | 4 | No | No | Control | 2 |
| Bthu_high | 17 | 5 | No | Yes | WW | 2 |
| Mixed_high | 23 | 5 | Yes | No | Aphids | 2 |
| Mixed_high | 23 | 6 | Yes | Yes | Aphids + WW | 2 |
| Mixed_high | 23 | 15 | No | No | Control | 2 |
| Mixed_high | 23 | 8 | No | Yes | WW | 2 |
| Athu_Bthu | 55 | 16 | Yes | No | Aphids | 2 |
| Athu_Bthu | 55 | 2 | Yes | Yes | Aphids + WW | 2 |
| Athu_Bthu | 55 | 3 | No | No | Control | 2 |
| Athu_Bthu | 55 | 1 | No | Yes | WW | 2 |
| Mixed_low | 67 | 11 | Yes | No | Aphids | 2 |
| Mixed_low | 67 | 2 | Yes | Yes | Aphids + WW | 2 |
| Mixed_low | 67 | 5 | No | No | Control | 2 |
| Mixed_low | 67 | 20 | No | Yes | WW | 2 |
| Chrys_Acet | 94 | 15 | Yes | No | Aphids | 2 |
| Chrys_Acet | 94 | 18 | Yes | Yes | Aphids + WW | 2 |
| Chrys_Acet | 94 | 10 | No | No | Control | 2 |
| Chrys_Acet | 94 | 14 | No | Yes | WW | 2 |
| Bthu_low | 101 | 9 | Yes | No | Aphids | 2 |
| Bthu_low | 101 | 18 | Yes | Yes | Aphids + WW | 2 |
| Bthu_low | 101 | 10 | No | No | Control | 2 |
| Bthu_low | 101 | 5 | No | Yes | WW | 2 |
| Bthu_high | 11 | 9 | Yes | No | Aphids | 3 |
| Bthu_high | 11 | 7 | Yes | Yes | Aphids + WW | 3 |
| Bthu_high | 11 | 1 | No | No | Control | 3 |
| Bthu_high | 11 | 10 | No | Yes | WW | 3 |
| Mixed_high | 23 | 9 | Yes | No | Aphids | 3 |
| Mixed_high | 23 | 1 | Yes | Yes | Aphids + WW | 3 |
| Mixed_high | 23 | 11 | No | No | Control | 3 |
| Mixed_high | 23 | 16 | No | Yes | WW | 3 |
| Athu_Bthu | 56 | 2 | Yes | No | Aphids | 3 |
| Athu_Bthu | 56 | 14 | Yes | Yes | Aphids + WW | 3 |
| Athu_Bthu | 56 | 12 | No | No | Control | 3 |
| Athu_Bthu | 56 | 9 | No | Yes | WW | 3 |
| Mixed_low | 68 | 12 | Yes | No | Aphids | 3 |
| Mixed_low | 68 | 15 | Yes | Yes | Aphids + WW | 3 |
| Mixed_low | 68 | 11 | No | No | Control | 3 |
| Mixed_low | 68 | 6 | No | Yes | WW | 3 |
| Chrys_Acet | 100 | 11 | Yes | No | Aphids | 3 |
| Chrys_Acet | 100 | 3 | Yes | Yes | Aphids + WW | 3 |
| Chrys_Acet | 100 | 8 | No | No | Control | 3 |
| Chrys_Acet | 100 | 10 | No | Yes | WW | 3 |
| Bthu_low | 103 | 11 | Yes | No | Aphids | 3 |
| Bthu_low | 103 | 1 | Yes | Yes | Aphids + WW | 3 |
| Bthu_low | 103 | 5 | No | No | Control | 3 |
| Bthu_low | 103 | 13 | No | Yes | WW | 3 |
| Bthu_high | 17 | 11 | Yes | No | Aphids | 4 |
| Bthu_high | 17 | 16 | Yes | Yes | Aphids + WW | 4 |
| Bthu_high | 17 | 3 | No | No | Control | 4 |
| Bthu_high | 17 | 10 | No | Yes | WW | 4 |
| Mixed_high | 30 | 19 | Yes | No | Aphids | 4 |
| Mixed_high | 30 | 8 | Yes | Yes | Aphids + WW | 4 |
| Mixed_high | 30 | 12 | No | No | Control | 4 |
| Mixed_high | 30 | 2 | No | Yes | WW | 4 |
| Athu_Bthu | 57 | 11 | Yes | No | Aphids | 4 |
| Athu_Bthu | 57 | 5 | Yes | Yes | Aphids + WW | 4 |
| Athu_Bthu | 57 | 2 | No | No | Control | 4 |
| Athu_Bthu | 57 | 12 | No | Yes | WW | 4 |
| Mixed_low | 64 | 8 | Yes | No | Aphids | 4 |
| Mixed_low | 64 | 9 | Yes | Yes | Aphids + WW | 4 |
| Mixed_low | 64 | 2 | No | No | Control | 4 |
| Mixed_low | 64 | 3 | No | Yes | WW | 4 |
| Chrys_Acet | 100 | 16 | Yes | No | Aphids | 4 |
| Chrys_Acet | 100 | 4 | Yes | Yes | Aphids + WW | 4 |
| Chrys_Acet | 100 | 13 | No | No | Control | 4 |
| Chrys_Acet | 100 | 2 | No | Yes | WW | 4 |
| Bthu_low | 104 | 15 | Yes | No | Aphids | 4 |
| Bthu_low | 104 | 10 | Yes | Yes | Aphids + WW | 4 |
| Bthu_low | 104 | 20 | No | No | Control | 4 |
| Bthu_low | 104 | 16 | No | Yes | WW | 4 |
| Bthu_high | 14 | 15 | Yes | No | Aphids | 5 |
| Bthu_high | 14 | 9 | Yes | Yes | Aphids + WW | 5 |
| Bthu_high | 14 | 4 | No | No | Control | 5 |
| Bthu_high | 14 | 3 | No | Yes | WW | 5 |
| Mixed_high | 23 | 20 | Yes | No | Aphids | 5 |
| Mixed_high | 23 | 19 | Yes | Yes | Aphids + WW | 5 |
| Mixed_high | 23 | 12 | No | No | Control | 5 |
| Mixed_high | 23 | 14 | No | Yes | WW | 5 |
| Athu_Bthu | 57 | 13 | Yes | No | Aphids | 5 |
| Athu_Bthu | 57 | 16 | Yes | Yes | Aphids + WW | 5 |
| Athu_Bthu | 57 | 1 | No | No | Control | 5 |
| Athu_Bthu | 57 | 10 | No | Yes | WW | 5 |
| Mixed_low | 64 | 5 | Yes | No | Aphids | 5 |
| Mixed_low | 64 | 18 | Yes | Yes | Aphids + WW | 5 |
| Mixed_low | 64 | 19 | No | No | Control | 5 |
| Mixed_low | 64 | 4 | No | Yes | WW | 5 |
| Chrys_Acet | 95 | 12 | Yes | No | Aphids | 5 |
| Chrys_Acet | 95 | 6 | Yes | Yes | Aphids + WW | 5 |
| Chrys_Acet | 95 | 18 | No | No | Control | 5 |
| Chrys_Acet | 95 | 13 | No | Yes | WW | 5 |
| Chrys_Acet | 95 | 20 | No | Yes | WW | 5 |
| Bthu_low | 103 | 3 | Yes | No | Aphids | 5 |
| Bthu_low | 103 | 10 | Yes | Yes | Aphids + WW | 5 |
| Bthu_low | 103 | 15 | No | No | Control | 5 |
| Bthu_low | 103 | 12 | No | Yes | WW | 5 |
| Bthu_high | 14 | 13 | Yes | No | Aphids | 6 |
| Bthu_high | 14 | 19 | Yes | Yes | Aphids + WW | 6 |
| Bthu_high | 14 | 6 | No | No | Control | 6 |
| Bthu_high | 14 | 20 | No | Yes | WW | 6 |
| Mixed_high | 30 | 17 | Yes | No | Aphids | 6 |
| Mixed_high | 30 | 20 | Yes | Yes | Aphids + WW | 6 |
| Mixed_high | 30 | 15 | No | No | Control | 6 |
| Mixed_high | 30 | 18 | No | Yes | WW | 6 |
| Athu_Bthu | 57 | 18 | Yes | No | Aphids | 6 |
| Athu_Bthu | 57 | 15 | Yes | Yes | Aphids + WW | 6 |
| Athu_Bthu | 57 | 6 | No | No | Control | 6 |
| Athu_Bthu | 57 | 19 | No | Yes | WW | 6 |
| Mixed_low | 68 | 14 | Yes | No | Aphids | 6 |
| Mixed_low | 68 | 13 | Yes | Yes | Aphids + WW | 6 |
| Mixed_low | 68 | 1 | No | No | Control | 6 |
| Mixed_low | 68 | 3 | No | Yes | WW | 6 |
| Chrys_Acet | 95 | 2 | Yes | No | Aphids | 6 |
| Chrys_Acet | 95 | 4 | Yes | Yes | Aphids + WW | 6 |
| Chrys_Acet | 95 | 1 | No | No | Control | 6 |
| Chrys_Acet | 95 | 3 | No | Yes | WW | 6 |
| Bthu_low | 104 | 3 | Yes | No | Aphids | 6 |
| Bthu_low | 104 | 5 | Yes | Yes | Aphids + WW | 6 |
| Bthu_low | 104 | 2 | No | No | Control | 6 |
| Bthu_low | 104 | 12 | No | Yes | WW | 6 |
| Bthu_high | 14 | 16 | Yes | No | Aphids | 7 |
| Bthu_high | 14 | 8 | Yes | Yes | Aphids + WW | 7 |
| Bthu_high | 14 | 18 | No | No | Control | 7 |
| Bthu_high | 14 | 10 | No | Yes | WW | 7 |
| Mixed_high | 21 | 13 | Yes | No | Aphids | 7 |
| Mixed_high | 21 | 2 | No | No | Control | 7 |
| Mixed_high | 21 | 4 | No | Yes | WW | 7 |
| Mixed_high | 30 | 13 | Yes | Yes | Aphids + WW | 7 |
| Athu_Bthu | 56 | 19 | Yes | No | Aphids | 7 |
| Athu_Bthu | 56 | 13 | Yes | Yes | Aphids + WW | 7 |
| Athu_Bthu | 56 | 11 | No | No | Control | 7 |
| Athu_Bthu | 56 | 17 | No | Yes | WW | 7 |
| Mixed_low | 68 | 18 | Yes | No | Aphids | 7 |
| Mixed_low | 68 | 9 | Yes | Yes | Aphids + WW | 7 |
| Mixed_low | 68 | 16 | No | No | Control | 7 |
| Mixed_low | 68 | 2 | No | Yes | WW | 7 |
| Chrys_Acet | 95 | 19 | Yes | No | Aphids | 7 |
| Chrys_Acet | 95 | 17 | Yes | Yes | Aphids + WW | 7 |
| Chrys_Acet | 95 | 5 | No | No | Control | 7 |
| Chrys_Acet | 95 | 8 | No | Yes | WW | 7 |
| Bthu_low | 104 | 9 | Yes | No | Aphids | 7 |
| Bthu_low | 104 | 7 | Yes | Yes | Aphids + WW | 7 |
| Bthu_low | 104 | 6 | No | No | Control | 7 |
| Bthu_low | 104 | 1 | No | Yes | WW | 7 |
| Bthu_high | 17 | 12 | Yes | No | Aphids | 8 |
| Bthu_high | 17 | 2 | Yes | Yes | Aphids + WW | 8 |
| Bthu_high | 17 | 9 | No | No | Control | 8 |
| Bthu_high | 17 | 8 | No | Yes | WW | 8 |
| Mixed_high | 30 | 10 | Yes | No | Aphids | 8 |
| Mixed_high | 30 | 16 | Yes | Yes | Aphids + WW | 8 |
| Mixed_high | 30 | 11 | No | No | Control | 8 |
| Mixed_high | 30 | 14 | No | Yes | WW | 8 |
| Athu_Bthu | 57 | 14 | Yes | No | Aphids | 8 |
| Athu_Bthu | 57 | 7 | Yes | Yes | Aphids + WW | 8 |
| Athu_Bthu | 57 | 9 | No | No | Control | 8 |
| Athu_Bthu | 57 | 3 | No | Yes | WW | 8 |
| Mixed_low | 67 | 1 | Yes | No | Aphids | 8 |
| Mixed_low | 67 | 3 | Yes | Yes | Aphids + WW | 8 |
| Mixed_low | 67 | 15 | No | No | Control | 8 |
| Mixed_low | 67 | 16 | No | Yes | WW | 8 |
| Chrys_Acet | 100 | 1 | Yes | No | Aphids | 8 |
| Chrys_Acet | 100 | 15 | Yes | Yes | Aphids + WW | 8 |
| Chrys_Acet | 100 | 9 | No | No | Control | 8 |
| Chrys_Acet | 100 | 5 | No | Yes | WW | 8 |
| Bthu_low | 101 | 4 | Yes | No | Aphids | 8 |
| Bthu_low | 101 | 12 | Yes | Yes | Aphids + WW | 8 |
| Bthu_low | 101 | 8 | No | No | Control | 8 |
| Bthu_low | 101 | 17 | No | Yes | WW | 8 |
| Bthu_high | 14 | 12 | Yes | No | Aphids | 9 |
| Bthu_high | 14 | 17 | Yes | Yes | Aphids + WW | 9 |
| Bthu_high | 14 | 1 | No | No | Control | 9 |
| Bthu_high | 14 | 11 | No | Yes | WW | 9 |
| Mixed_high | 21 | 5 | Yes | No | Aphids | 9 |
| Mixed_high | 21 | 1 | Yes | Yes | Aphids + WW | 9 |
| Mixed_high | 21 | 6 | No | No | Control | 9 |
| Mixed_high | 21 | 3 | No | Yes | WW | 9 |
| Athu_Bthu | 55 | 5 | Yes | No | Aphids | 9 |
| Athu_Bthu | 55 | 20 | Yes | Yes | Aphids + WW | 9 |
| Athu_Bthu | 55 | 6 | No | No | Control | 9 |
| Athu_Bthu | 55 | 11 | No | Yes | WW | 9 |
| Mixed_low | 67 | 12 | Yes | No | Aphids | 9 |
| Mixed_low | 67 | 18 | Yes | Yes | Aphids + WW | 9 |
| Mixed_low | 67 | 10 | No | No | Control | 9 |
| Mixed_low | 67 | 7 | No | Yes | WW | 9 |
| Chrys_Acet | 94 | 2 | Yes | No | Aphids | 9 |
| Chrys_Acet | 94 | 1 | Yes | Yes | Aphids + WW | 9 |
| Chrys_Acet | 94 | 12 | No | No | Control | 9 |
| Chrys_Acet | 94 | 16 | No | Yes | WW | 9 |
| Bthu_low | 101 | 16 | Yes | No | Aphids | 9 |
| Bthu_low | 101 | 7 | Yes | Yes | Aphids + WW | 9 |
| Bthu_low | 101 | 14 | No | No | Control | 9 |
| Bthu_low | 101 | 3 | No | Yes | WW | 9 |
| Bthu_high | 11 | 6 | Yes | No | Aphids | 10 |
| Bthu_high | 11 | 15 | Yes | Yes | Aphids + WW | 10 |
| Bthu_high | 11 | 16 | No | No | Control | 10 |
| Bthu_high | 11 | 12 | No | Yes | WW | 10 |
| Mixed_high | 21 | 10 | Yes | No | Aphids | 10 |
| Mixed_high | 21 | 11 | Yes | Yes | Aphids + WW | 10 |
| Mixed_high | 21 | 7 | No | No | Control | 10 |
| Mixed_high | 21 | 17 | No | Yes | WW | 10 |
| Athu_Bthu | 56 | 3 | Yes | No | Aphids | 10 |
| Athu_Bthu | 56 | 20 | Yes | Yes | Aphids + WW | 10 |
| Athu_Bthu | 56 | 4 | No | No | Control | 10 |
| Athu_Bthu | 56 | 18 | No | Yes | WW | 10 |
| Mixed_low | 64 | 1 | Yes | No | Aphids | 10 |
| Mixed_low | 64 | 14 | Yes | Yes | Aphids + WW | 10 |
| Mixed_low | 64 | 20 | No | No | Control | 10 |
| Mixed_low | 64 | 6 | No | Yes | WW | 10 |
| Chrys_Acet | 100 | 17 | Yes | No | Aphids | 10 |
| Chrys_Acet | 100 | 14 | Yes | Yes | Aphids + WW | 10 |
| Chrys_Acet | 100 | 6 | No | No | Control | 10 |
| Chrys_Acet | 100 | 20 | No | Yes | WW | 10 |
| Bthu_low | 101 | 11 | No | Yes | WW | 10 |
| Bthu_low | 103 | 4 | Yes | No | Aphids | 10 |
| Bthu_low | 103 | 6 | Yes | Yes | Aphids + WW | 10 |
| Bthu_low | 103 | 2 | No | No | Control | 10 |

**Table 6** R-CODE FOR ALL MODELS

| **Table** | **Model** | **R-code** |
| --- | --- | --- |
| 1 | A | Lmer(sqrt(Final aphid colony size) ~ Belowground (yes/no) * Chemotype * Day  + (1\|Block) + (1\|Daughter) + (1\|Day/Daughter) |
| 1 | B | Lmer(sqrt(Aphid colony size) ~ Retrieved wireworm larvae * Chemotype * Day  + (1\|Block) + (1\|Daughter) + (1\|Day/Daughter) |
| 2 | A | Lmer(sqrt(Final aphid colony size) ~ Belowground (yes/no) + Terpenoid evenness + Terpenoid richness + Terpenoid concentration  + (1\|Block) |
| 2 | B | Lmer(sqrt(Final aphid colony size) ~ Retrieved wireworm larvae + Terpenoid evenness + Terpenoid richness + Terpenoid concentration  + (1\|Block) |
| 3 | Plant dry weight | Lmer(Dry weight ~ *Coloradoa tanacetina* + Aboveground * Belowground * Chemotype  + (1\|Block) + (1\|Daughter) |
| 3 | Plant height | Lmer(Plant height ~ *Coloradoa tanacetina* + Aboveground * Belowground * Chemotype  + (1\|Block) + (1\|Daughter) |
| 3 | Chlorophyll content | Lmer(Chlorophyll content ~ *Coloradoa tanacetina* + Aboveground * Belowground * Chemotype  + (1\|Block) + (1\|Daughter) |
| 7 | Chemotype | Glmer(Aphid survival ~ Chemotype  + (1\|Block) + (1\|Daughter),  Family = binomial |
| 7 | Daughters | Glmer(Aphid survival ~ (1\|Chemotype/Daughter)  + (1\|Block),  Family = binomial |
| 8 | A, B | See Table 1; Plants with zero aphids were included into the dataset |
| 9 | A, B | See Table 2; Plants with zero aphids were included into the dataset |
| 10 | A | Lmer(sqrt(Final aphid colony size) ~ *Coloradoa tanacetina* + Chemotype * Belowground (yes/no)  + (1\|Block) + (1\|Daughter) |
| 10 | B | Lmer(sqrt(Final aphid colony size) ~ *Coloradoa tanacetina* + Chemotype * Retrieved wireworm larvae  + (1\|Block) + (1\|Daughter) |
| 11 |  | Lmer(sqrt(*Coloradoa tanacetina*) ~ Chemotype * sqrt(Final aphid colony size)  + (1\|Block) + (1\|Daughter) |

**Table 7** OUTPUT FROM A MIXED LINEAR MODEL FOR *M. TANACETARIA* SURVIVAL IN PLANTS THAT WERE ASSIGNED APHID TREATMENT, USING CHEMOTYPE (MODEL A) OR DAUGHTER NESTED WITHIN CHEMOTYPE (MODEL B) OR BG TREATMENT (MODEL C) OR NUMBER OF RETREIVED WIREWORM LARVAE (MODEL D) AS FIXED EFFECTS AND THE DAUGHTER (NOT IN MODEL B) AND THE BLOCK AS RANDOM EFFECT

|  |  | **d.f.** | **χ^2^ (p-value)** |
| --- | --- | --- | --- |
| **Model A** | Chemotype | 5 | 7.36 (0.195) |
| **Model B** | Daughter | 1 | **11.30 (<0.001)** |
| **Model C** | BG | 1 | 0.08 (0.784) |
| **Model D** | Wireworm Larvae | 1 | 0.01 (0.916) |


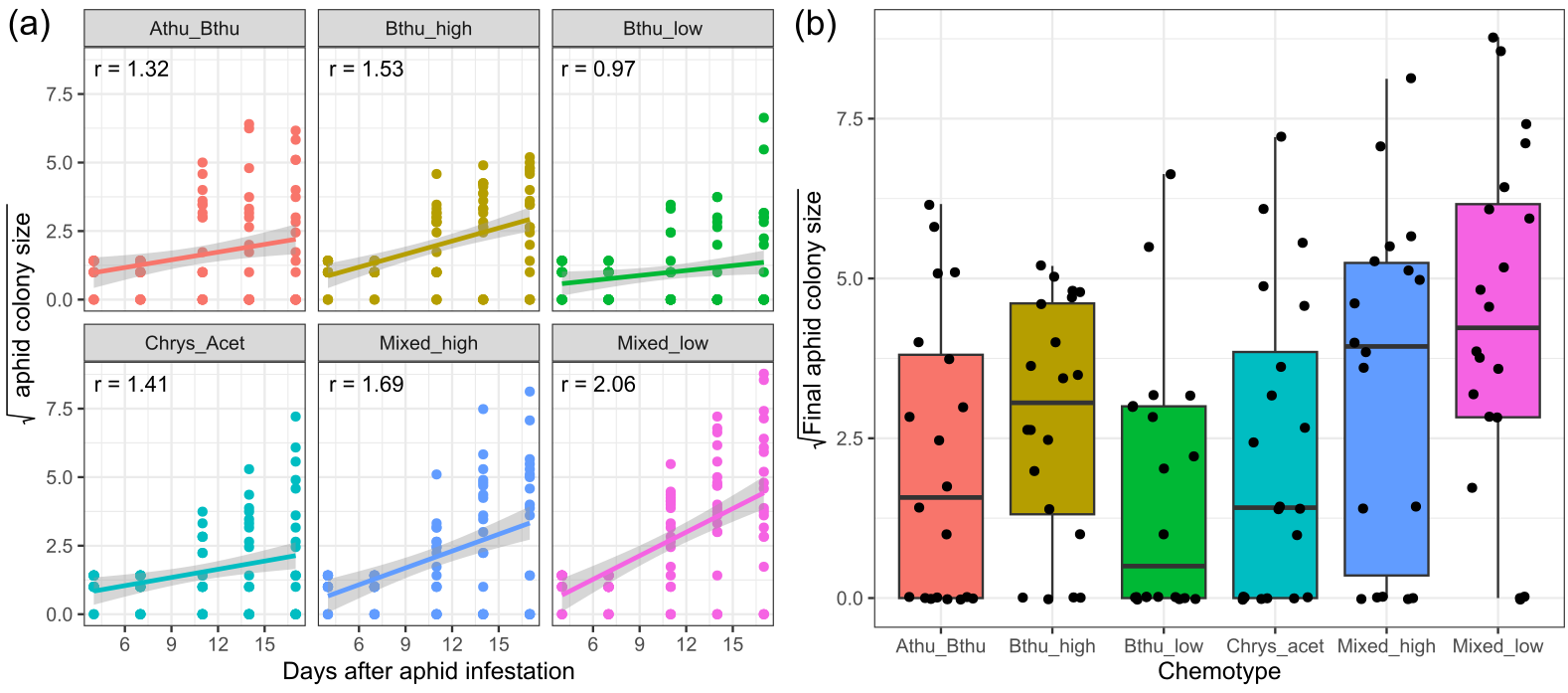


**Fig. 6** (a) Square root-transformed *M. tanacetaria* colony size over time in days after aphid infestation, across chemotypes. R-values represent the slope of fitted lines. (b) Final aphid colony size at the time of the experimental harvest for different tansy chemotypes. Boxes represent the variation in data, where the lower hinge corresponds to the first quartile (25th percentile) and the upper hinge depicts the third quartile (75th percentile). Whiskers indicate the 5% and 95% percentiles; solid lines within boxes represent the medians. Black dots indicate individual sample values. The six chemotypes are depicted in different colours for convenience

**Table 8** OUTPUT FROM A MIXED LINEAR MODEL FOR M. TANACETARIA COLONY SIZE OVER TIME, USING EITHER BELOWGROUND HERBIVORY TREATMENT (BELOWGROUND) (MODEL A) OR THE NUMBER OF RETRIEVED WIREWORM LARVAE (WIREWORM LARVAE) (MODEL B), AND DAY AND CHEMOTYPE, AND THE INTERACTION TERMS AS FIXED EFFECTS. IN BOTH MODELS, BLOCK, DAUGHTER, AND INDIVIDUAL ID (NESTED WITHIN DAY) WERE USED AS RANDOM EFFECTS

| **Model A** | **d.f.** | **χ^2^ (p-value)** | **Model B** | **d.f.** | **χ^2^ (p-value)** |
| --- | --- | --- | --- | --- | --- |
| Belowground | 1 | 2.04 (0.153) | Wireworm larvae | 3 | 2.95 (0.399) |
| Chemotype | 5 | 7.33 (0.197) | Chemotype | 5 | 6.67 (0.246) |
| Day | 1 | **86.01 (<0.001)** | Day | 1 | **84.71 (<0.001)** |
| Belowground * Chemotype | 5 | 3.06 (0.691) | Wireworm larvae * Chemotype | 14 | 13.30 (0.503) |
| Belowground * Day | 1 | 0.10 (0.751) | Wireworm larvae * Day | 3 | 4.03 (0.258) |
| Chemotype * Day | 5 | **22.85 (0.001)** | Chemotype * Day | 5 | **17.34 (0.004)** |
| Belowground * Chemotype * Day | 5 | 3.62 (0.606) | Wireworm larvae * Chemotype * Day | 14 | 9.02 (0.830) |

**Table 9** OUTPUT FROM A MIXED LINEAR MODEL FOR FINAL M. TANACETARIA COLONY SIZE, USING BELOWGROUND HERBIVORY TREATMENT (BG) (MODEL A) OR THE NUMBER OF RETRIEVED WIREWORM LARVAE (MODEL B), AND TERPENOID RICHNESS, TERPENOID EVENNESS AND TOTAL TERPENOID CONCENTRATION CALCULATED BASED ON THE TERPENOID PROFILE OF THE 18 DAUGHTER PLANTS (THREE FOR EACH OF THE SIX CHEMOTYPES) AS FIXED EFFECTS AND THE BLOCK AS RANDOM EFFECT

| **Model A** | **d.f.** | **χ^2^ (p-value)** | **Model B** | **d.f.** | **χ^2^ (p-value)** |
| --- | --- | --- | --- | --- | --- |
| BG | 1 | 0.52 (0.469) | Wireworm larvae | 3 | **8.56 (0.036)** |
| Evenness | 1 | **5.77 (0.016)** | Evenness | 1 | *3.82 (0.051)* |
| Richness | 1 | 0.00 (0.990) | Richness | 1 | 0.00 (0.951) |
| Concentration | 1 | 2.00 (0.157) | Concentration | 1 | 2.18 (0.140) |


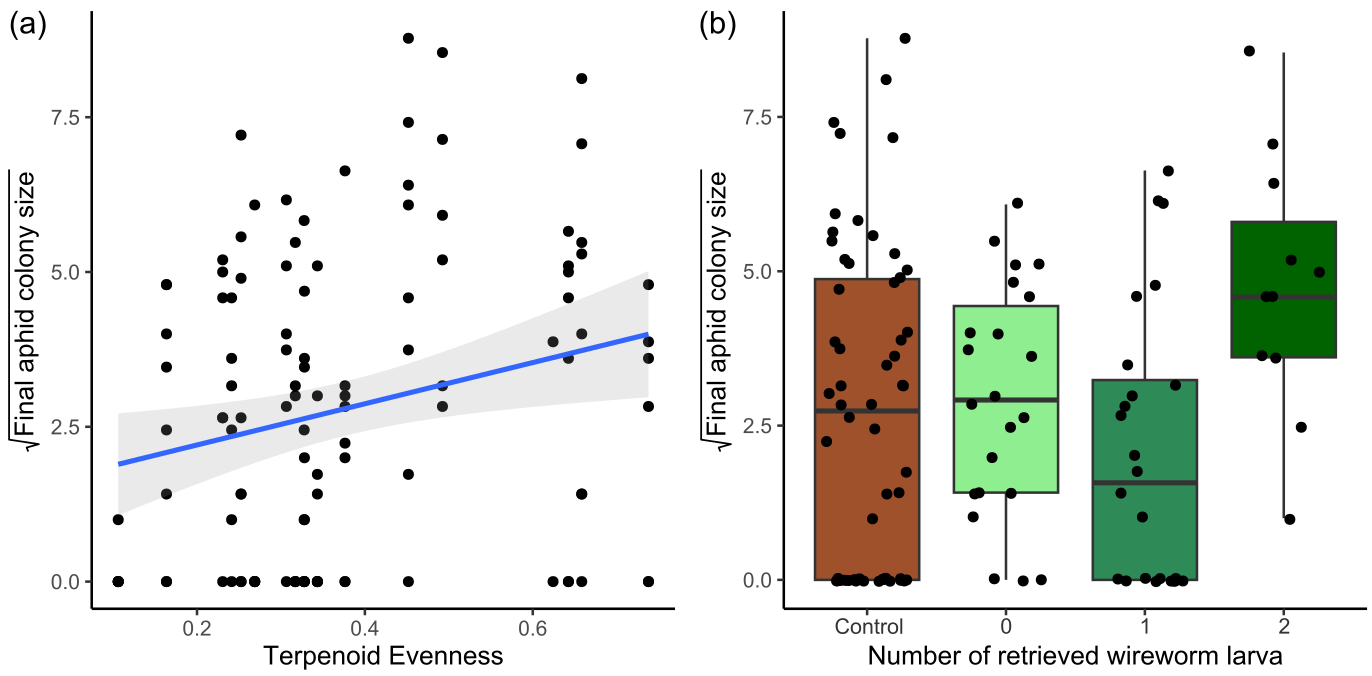


**Fig. 7** (a) Square root-transformed *M. tanacetaria* colony size on plants differing in leaf terpenoid evenness. The linear trendline depicts average predicted values based on a linear model, and the shaded area depicts the 95% confidence interval. (b) Box plots visualizing square root-transformed *M. tanacetaria* colony size on plants with no added wireworms, compared to plants on which 0, 1 or 2 wireworm larvae were retrieved after the harvest. Boxes represent the variation in data, where the lower hinge corresponds to the first quartile (25th percentile) and the upper hinge depicts the third quartile (75th percentile). Whiskers indicate the 5% and 95% percentiles; solid lines within boxes represent the medians. Black dots indicate individual sample values

**Table 10** OUTPUT FROM A MIXED LINEAR MODEL FOR THE M. TANACETARIA COLONY SIZE (SQUARE ROOT TRANSFORMED), TAKING C. TANACETINA ABUNDANCE, CHEMOTYPE, AND EITHER BELOWGROUND HERBIVORY TREATMENT (BG) (MODEL A) OR THE NUMBER OF RETRIEVED WIREWORM LARVAE (MODEL B) AND THE INTERPLAY OF CHEMOTYPE AND BELOWGROUND TREATMENT OR CHEMOTYPE AND THE NUMBER OF WIREWORM LARVAE AS FIXED EFFECTS, AND THE DAUGHTER AND BLOCK AS RANDOM EFFECT INTO ACCOUNT

| **Model A** | **d.f.** | **χ^2^ (p-value)** | **Model B** | **d.f.** | **χ^2^ (p-value)** |
| --- | --- | --- | --- | --- | --- |
| *C. tanacetina* | 1 | 2.09 (0.148) | *C. tanacetina* | 1 | 1.53 (0.216) |
| Chemotype | 5 | **14.66 (0.012)** | Chemotype | *5* | *9.40 (0.094)* |
| BG | 1 | 0.17 (0.683) | Wireworm larvae | 3 | 5.79 (0.122) |
| Chemotype * BG | 5 | 3.28 (0.657) | Chemotype * Wireworm larvae | 14 | 9.45 (0.801) |

**Table 11** OUTPUT FROM A MIXED LINEAR MODEL FOR THE C. TANACETINA ABUNDANCE (SQUARE ROOT TRANSFORMED), TAKING M. TANACETARIA COLONY SIZE (SQUARE ROOT TRANSFORMED), CHEMOTYPE, AND THE INTERPLAY OF CHEMOTYPE AND M. TANACETARIA AS FIXED EFFECTS, AND THE DAUGHTER AND BLOCK AS RANDOM EFFECT INTO ACCOUNT

|  | **d.f.** | **χ^2^ (p-value)** |
| --- | --- | --- |
| Chemotype | 5 | 1.49 (0.914) |
| *M. tanacetaria* | 1 | 2.21 (0.137) |
| Chemotype * *M. tanacetaria* | 5 | 7.66 (0.176) |


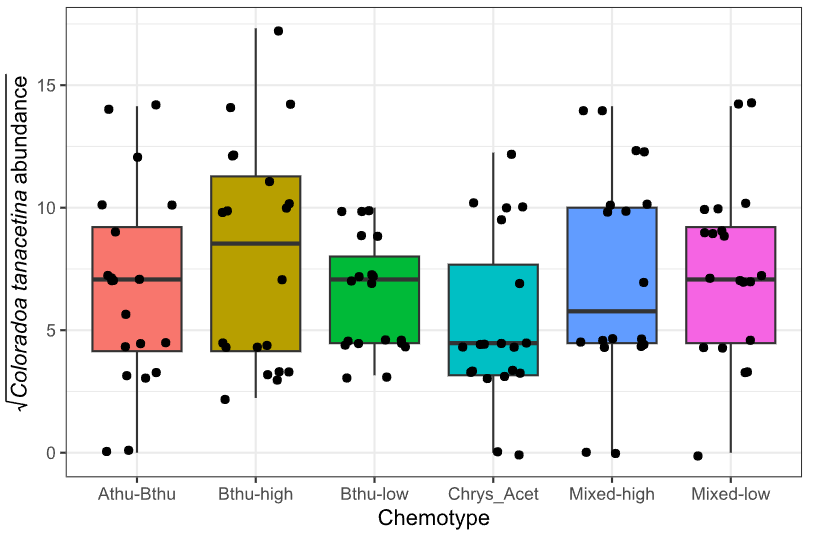


**Fig. 8** Estimated *C. tanacetina* numbers for different tansy chemotypes three days after the final count of *M. tanacetaria*. Boxes represent the variation in data, where the lower hinge corresponds to the first quartile (25th percentile) and the upper hinge depicts the third quartile (75th percentile). Whiskers indicate the 5% and 95% percentiles; solid lines within boxes represent the medians. Black dots indicate individual sample values. The six chemotypes are depicted in different colours for convenience


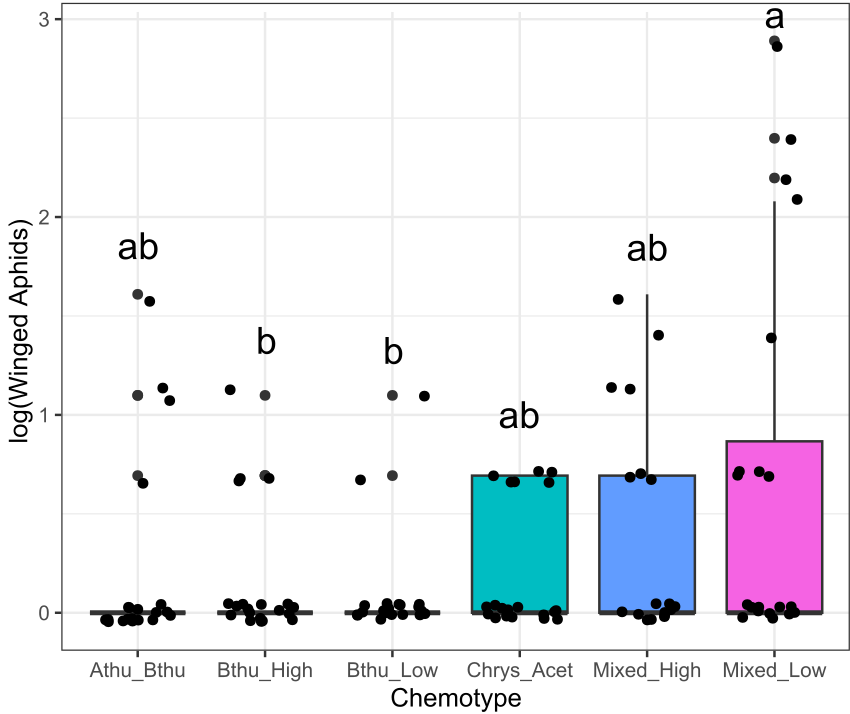


**Fig. 9** Numbers of winged adult aphids (alates) for different tansy chemotypes three days after the final count of *M. tanacetaria*. Boxes represent the variation in data, where the lower hinge corresponds to the first quartile (25th percentile) and the upper hinge depicts the third quartile (75th percentile). Whiskers indicate the 5% and 95% percentiles; solid lines within boxes represent the medians. Black dots indicate individual sample values. The six chemotypes are depicted in different colours for convenience. Letters depict statistical significance based on posthoc Tukey tests.


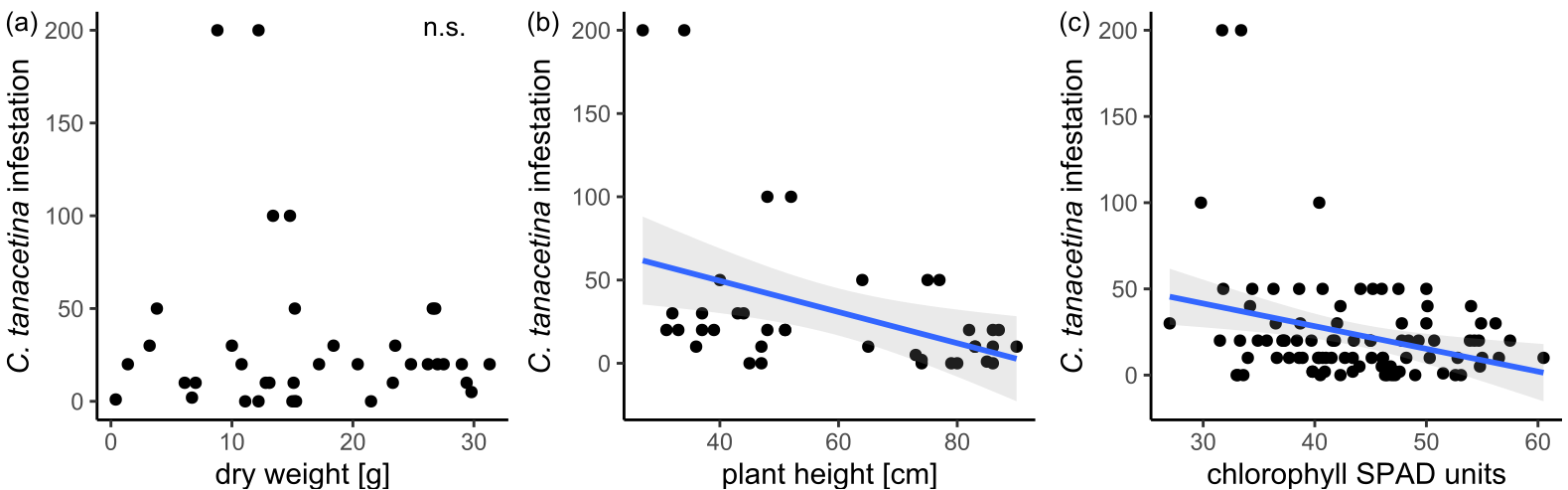


**Fig. 10** (a) Infestation of *Coloradoa tanacetina* in relationship to plant dry weight (g). (b) Infestation of *Coloradoa tanacetina* in relationship to plant height (cm). (c) Infestation of *Coloradoa tanacetina* in relationship to plant chlorophyll (SPAD units). The linear trendline in (b) and (c) depicts average predicted values based on a linear model, and the shaded area depicts the 95% confidence interval
